# Supplementary material for: Self-spinning filaments for autonomously linked microfibers
Source: Nat Commun. 2023 Feb 4;14:625. doi: 10.1038/s41467-023-36355-w (PMC9899204; doi:10.1038/s41467-023-36355-w)
Supplement: Supplementary file 1 — Supplementary Information [file 41467_2023_36355_MOESM1_ESM.pdf]

Supplementary Information for:

Self-Spinning Filaments for Autonomously Linked Microfibers

Authors: Dylan M. Barber[1], Todd Emrick[1]*, Gregory M. Grason[1]*, and Alfred J. Crosby[1]*

Affiliations: [1]Polymer Science and Engineering Department; University of Massachusetts Amherst; Amherst, MA 01003-9263, USA

Inventory of Supplementary Information

Supplementary Figures 1 to 23

Supplementary Notes 1 to 4

Supplementary Movies 1 to 12

Supplementary Figures

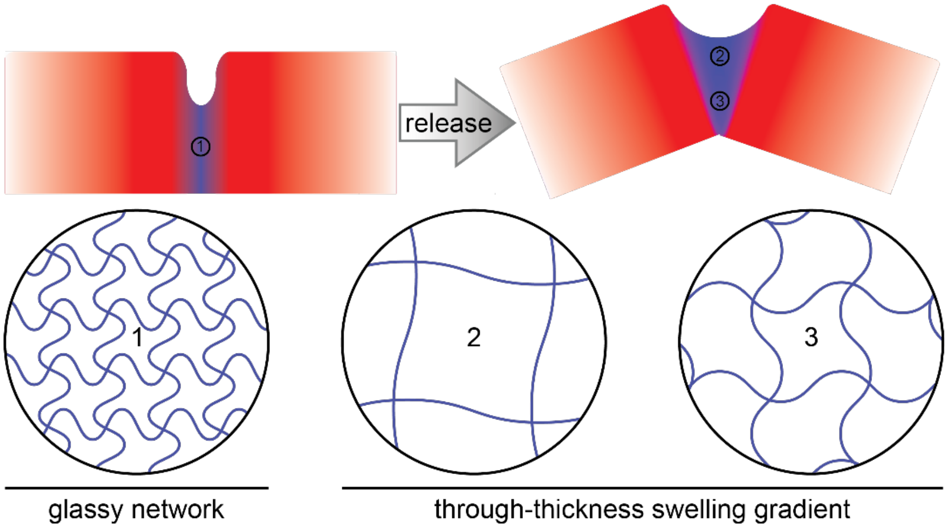

Supplementary Fig. 1. Schematic depiction of gradient swelling to afford bottom-face-in photocrease bending upon release into aqueous solution.

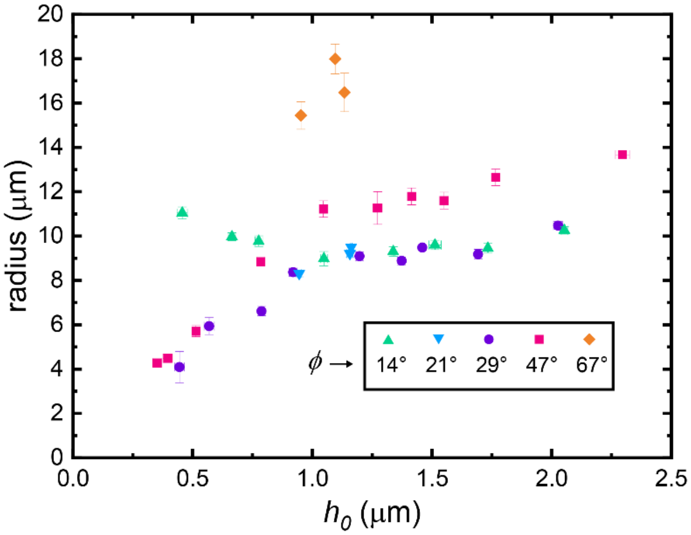

Supplementary Fig. 2. Helix radius as a function of as-printed thickness h_0 and photocrease tilt angle ϕ. Error bars in radius represent 1 standard deviation of the sample summed in quadrature with rms displacement from the fitted helix. Error bars in h_0 represent 1 standard deviation of the sample.

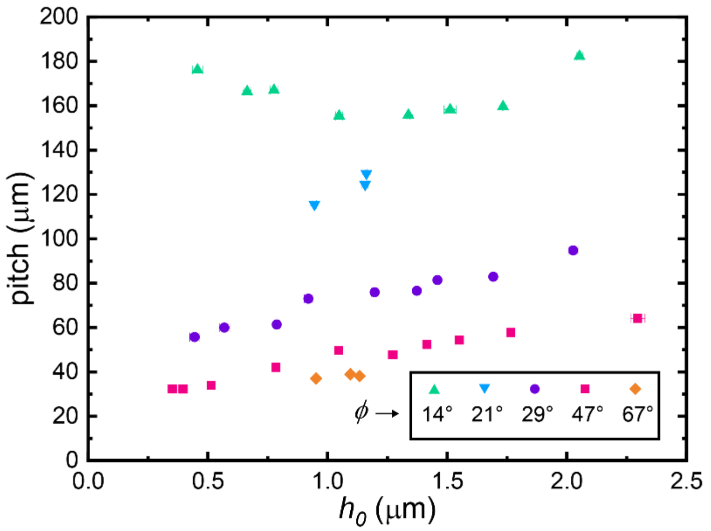

Supplementary Fig. 3. Helix pitch as a function of as-printed thickness h_0 and photocrease tilt angle ϕ. Error bars in pitch represent 1 standard deviation of the sample summed in quadrature with rms displacement from the fitted helix. Error bars in h_0 represent 1 standard deviation of the sample.

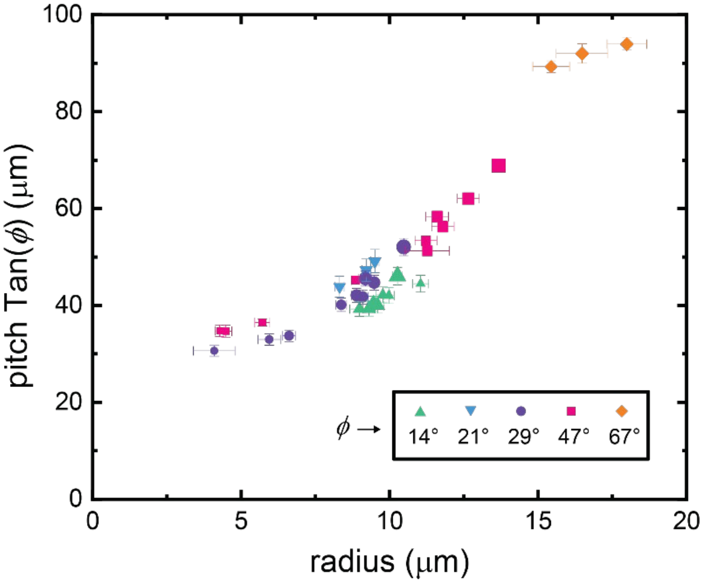

Supplementary Fig. 4. Data of Fig. 3b plotted *p* (Tan(ϕ)) as a function of helical radius to collapse onto a single trend. As in Fig. 3b, data point size corresponds to h_0, spanning 350-2300 nm. Error bars in radius represent 1 standard deviation of the sample summed in quadrature with rms displacement from the fitted helix. Error in pitch was calculated the same way, then added in quadrature to the standard deviation of Tan(ϕ) to afford the error bars in *p* (Tan(ϕ)).

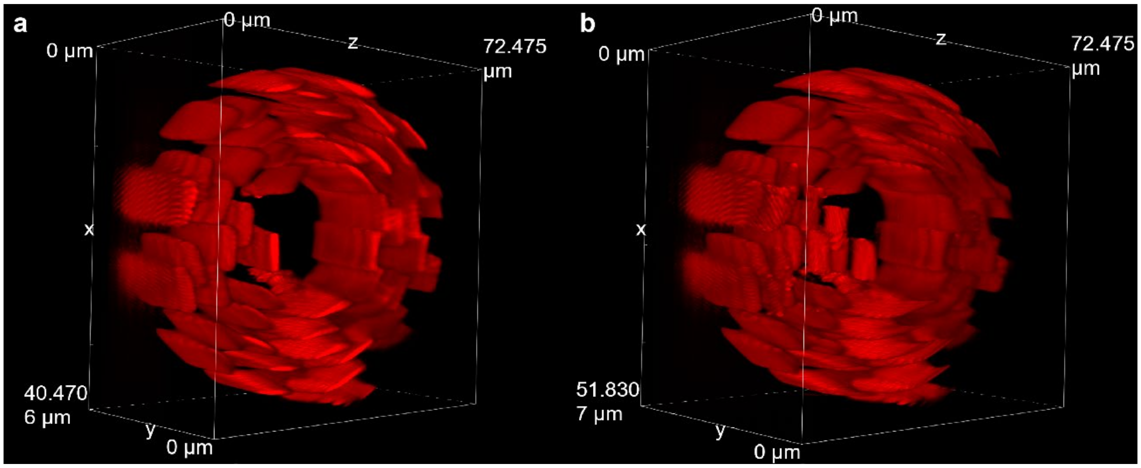

Supplementary Fig. 5. Achiral roll from ϕ = 90°. a, the 3D-reconstructed z-stack with in-plane crop to elucidate individual segments shown in Fig 3G; **b**, the same data set in a ‘raw’ and uncropped form.

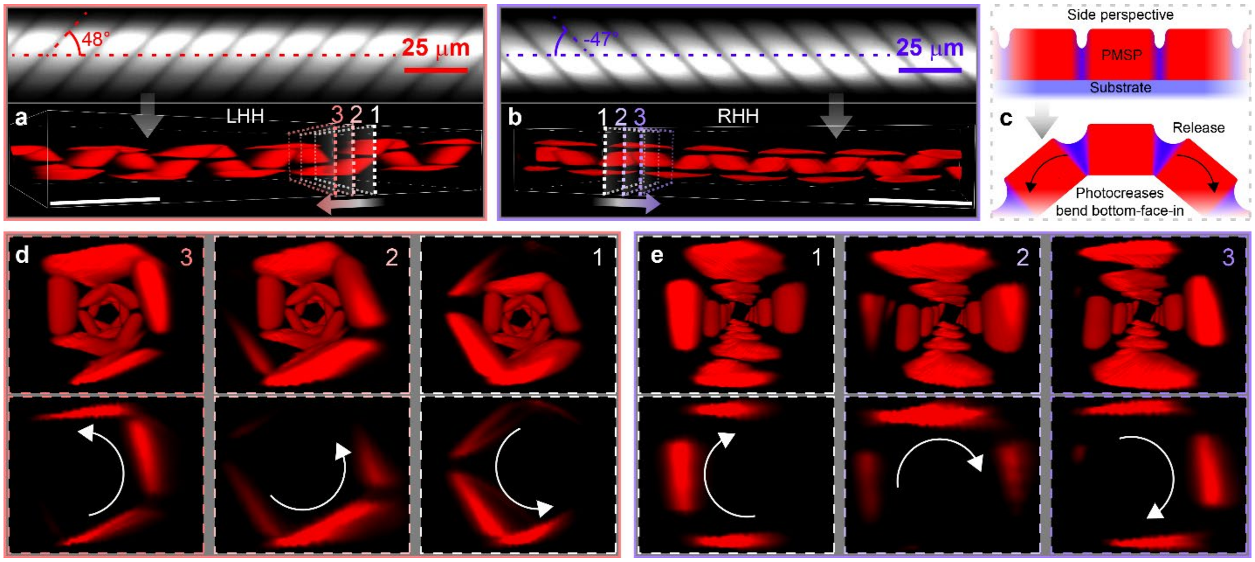

Supplementary Fig. 6. Programmed chirality. MSPs with ϕ = 48° (**a**) and -47° (**b**) before (top, conventional fluorescence) and after (bottom, confocal fluorescence) release to afford (**a**) left-handed or (**b**) right handed helices (50 μm scale bar); sequential cross-sections and viewing angle are denoted by dashed lines and arrows, respectively; **c**, schematic representation (side perspective) of a PMSP before (top) and after (bottom) release, showing that photocreases bend 'bottom-face-in;' axial cross-sections of the helices in panels **a** and **b** respectively lead to counterclockwise (**d**) and clockwise (**e**) rotation to confirm left- and right-handed chirality. The upper image includes all voxels in the background volume, revealing helical coils; the bottom cross-section includes only voxels in the cross-sectional plane.

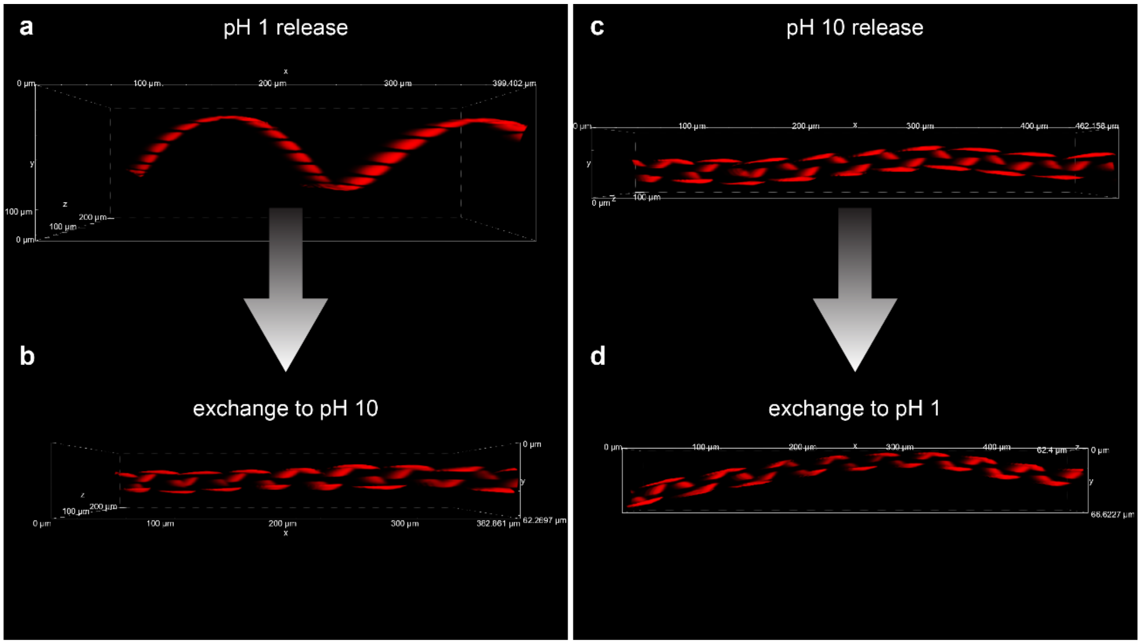

Supplementary Fig. 7. pH-dependent coiling. Photocreased MSPs with ϕ ~ 45° and h_0 ~ 1 μm after: **a**, release into pH 1 buffer, then **b**, exchange into pH 10 buffer; **c**, release into pH 10 buffer, then **d**, exchange into pH 1 buffer.

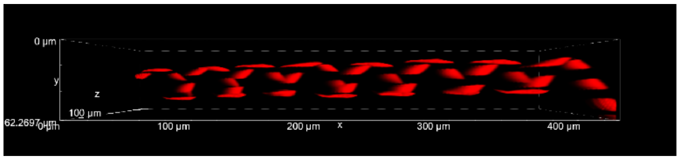

Supplementary Fig. 8. coiling in the presence of surfactant. A photocreased MSP with ϕ ~ 45° and h_0 ~ 1 μm released into a pH 8 buffer with 3 mM SDS to reduce polymer-water interfacial tension.

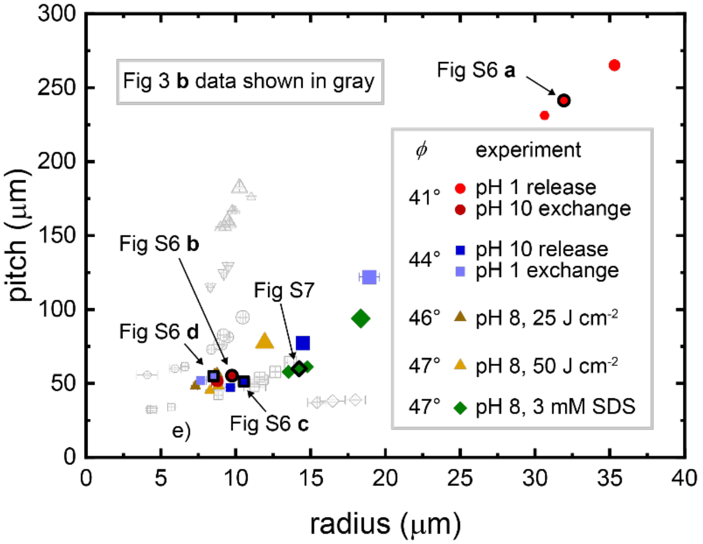

Supplementary Fig. 9. Helix dimensions under special conditions, including release into pH 1 buffer (red points) and exchange to pH 10 (maroon); release into pH 10 buffer (dark blue) and exchange to pH 1 (light blue); patterning at reduced UV doses of 25 (brown) and 50 J cm^{-2} (yellow); release into a solution of 3 mM SDS (green) to reduce interfacial energy. Points with black borders correspond to the 5 helices in Supplementary Figures 7 and 8. Error bars represent 1 standard deviation of the sample summed in quadrature with rms displacement from the fitted helix.

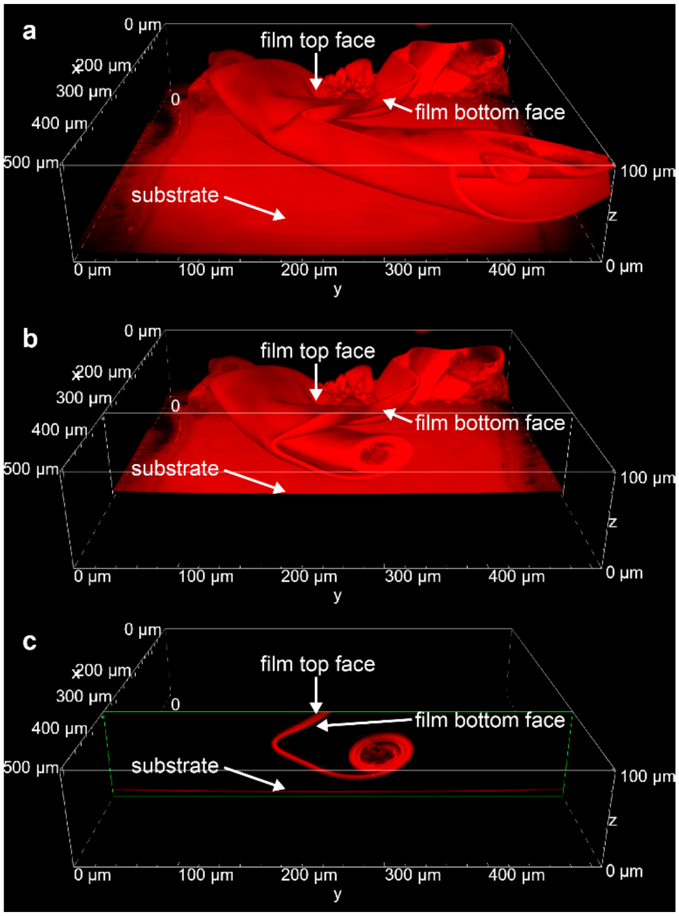

Supplementary Fig. 10. Bottom-face-in rolling of a deprotected film. a, 3D confocal z-stack of a deprotected film after release in pH 8 buffer solution. The bottom face of the film faces toward the substrate; **b**, the same z-stack with a cross-sectional slice revealing a rolled tube with bottom-face-in curvature; **c**, the same cross-sectional slice excluding all background voxels.

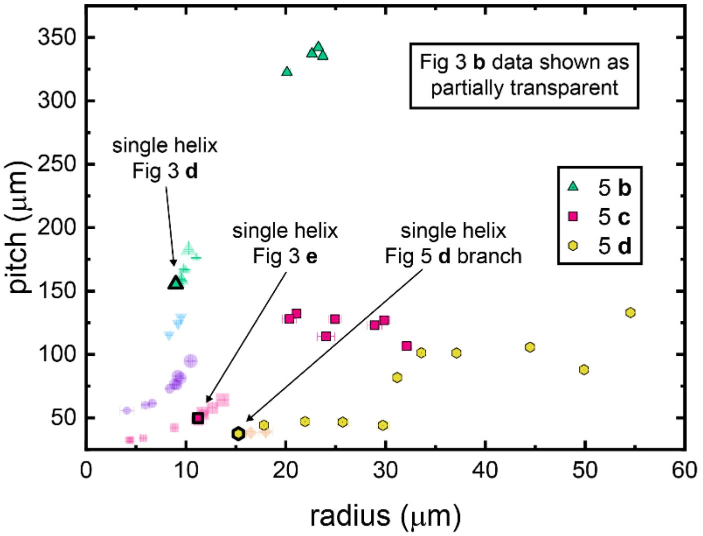

Supplementary Fig. 11. Pitch and radius in bundled MSPs. Pitch and radius of best fit applied to centroid data for the 4, 7, and 10 MSPs bundled in Fig 5**b**-**d**, plotted against the data of Fig 3**b** (partially transparent). Note that the Fig 5**d** data is taken from a 60 μm section of the bundle. Error bars in pitch and radius represent the rms axial and radial (respectively) displacement from the fitted helix added in quadrature to 1 standard deviation of the sample in pitch and radius (respectively).

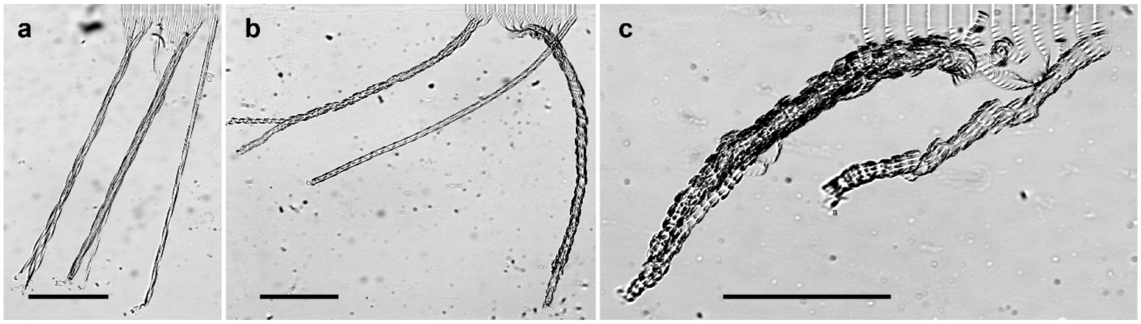

Supplementary Fig. 12. curvature in MSP bundles as a function of ϕ. Bundled MSPs with arc length ~ 3 mm where ϕ = 14° (**a**), showing negligible change in helix axis direction in 3 bundles of ~1.8 mm length, 46° (**b**), showing curvature of the helix axis in 3 bundles of 1.8-2 mm length, and 76° (**c**), with a curved helix axis in 2 helices of length 1-1.3 mm. Scale bars 500 μm.

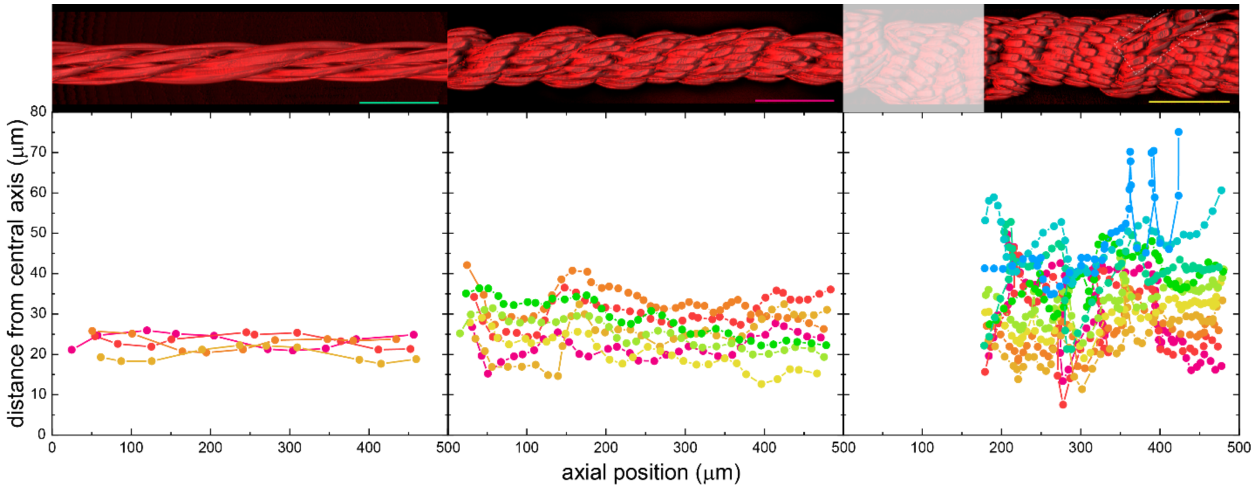

Supplementary Fig. 13. Radial migration in bundled MSPs. Confocal micrographs (top, scale bars 100 μm) and plots of segment centroid distance from the central axis (i.e. radius) as a function of position along the bundle length (bottom). The data sets correspond to the bundles of 4 MSPs in Fig 5b (left), 7 MSPs in Fig 5c (center), and 10 MSPs in Fig 5d (right). Note that the rightmost data set has been truncated to approximate a straight helix axis.

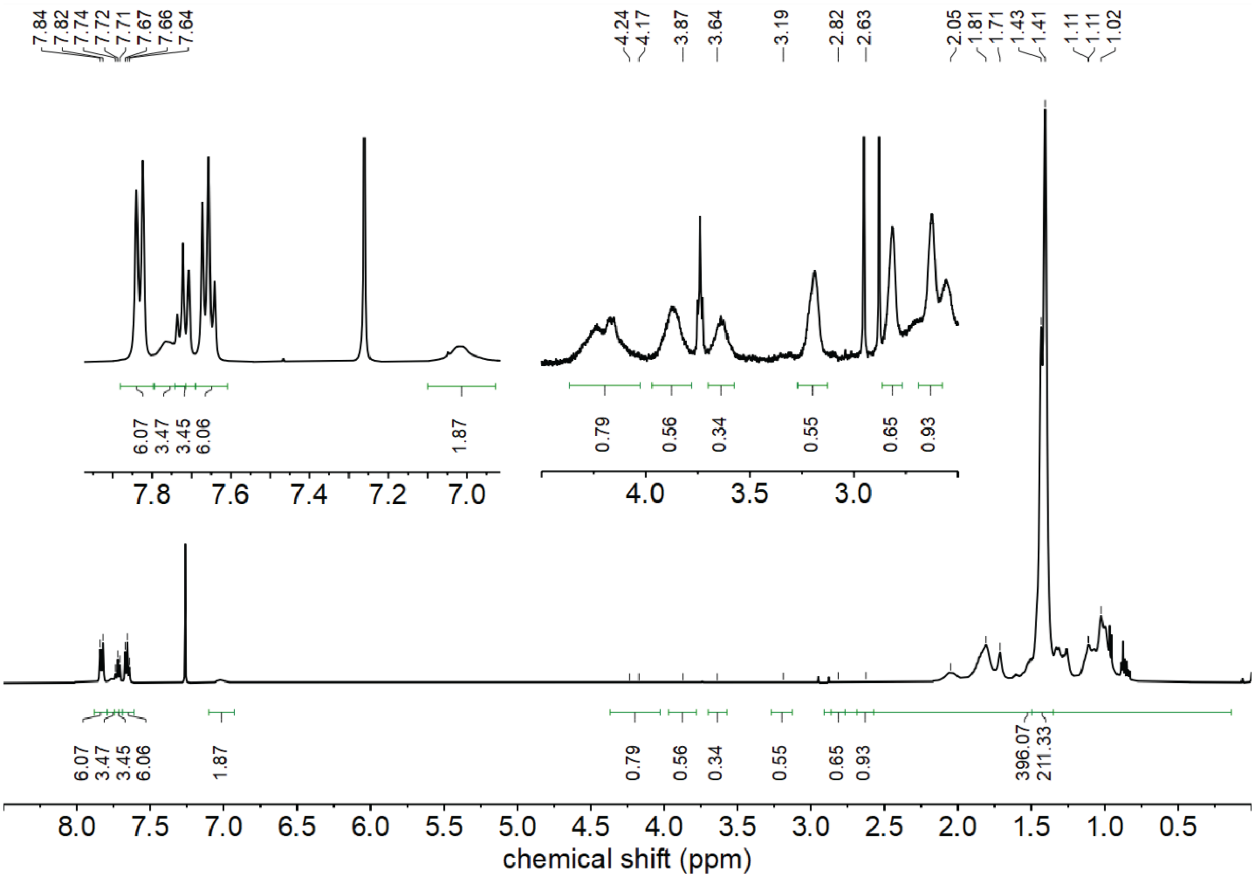

Supplementary Fig. 14. ^{1}H NMR spectrum of copolymer **1**.

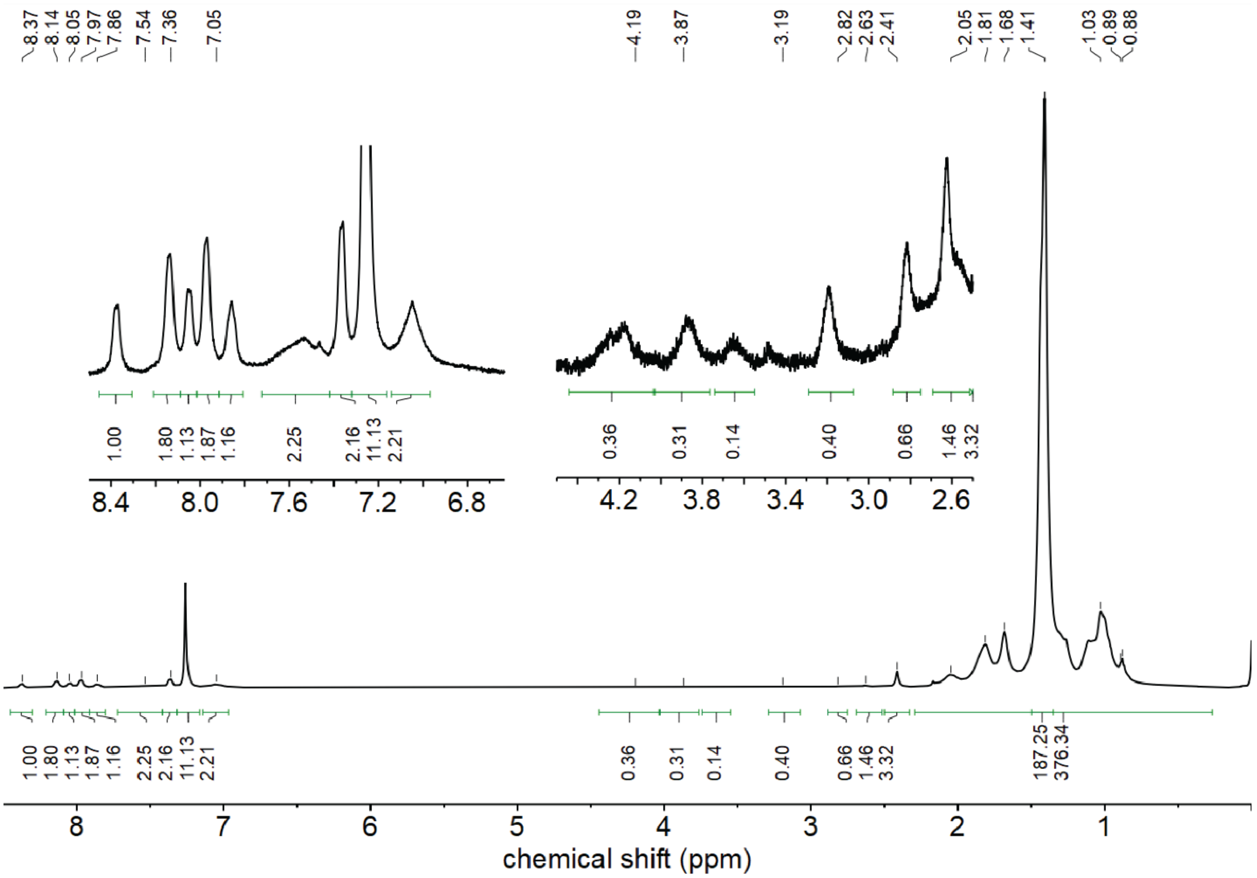

Supplementary Fig. 15. ^{1}H NMR spectrum of copolymer **2**.

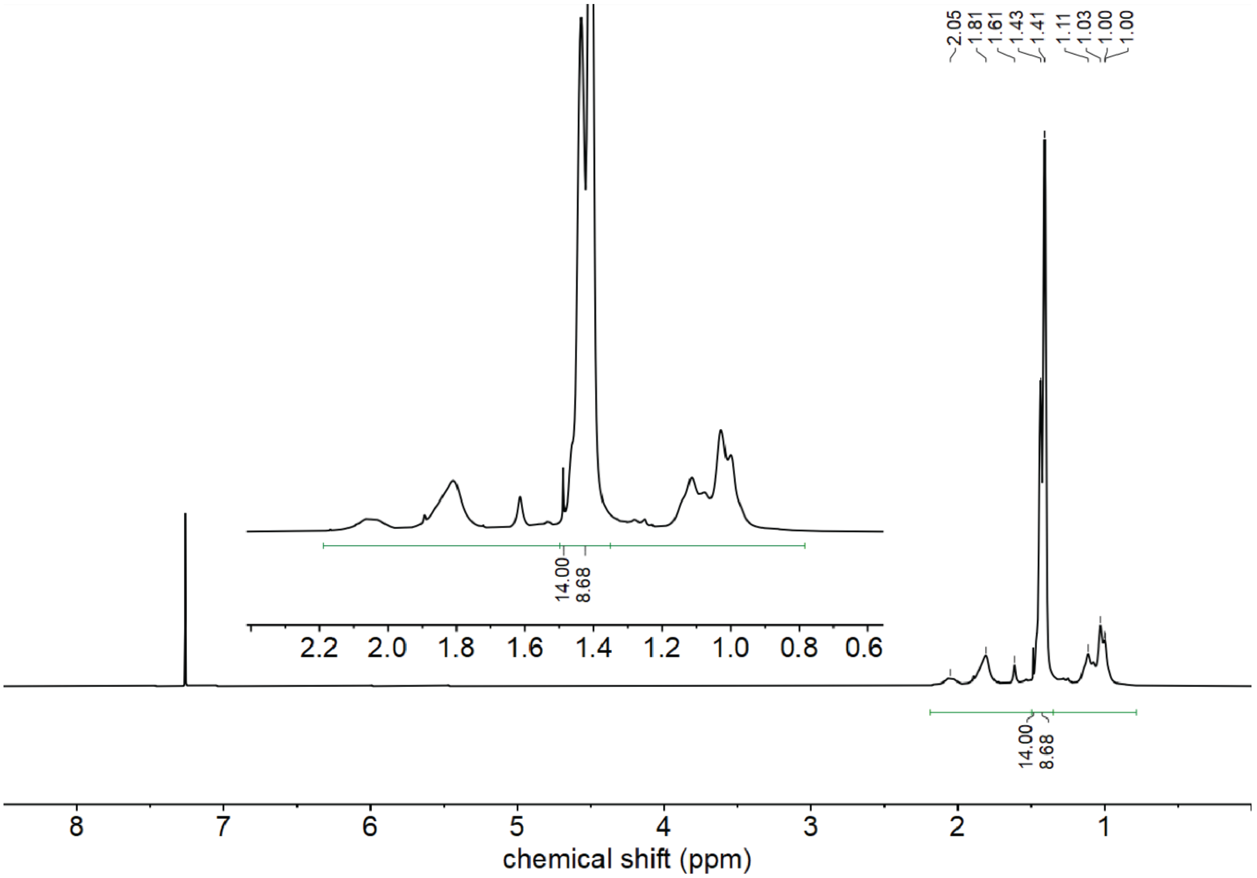

Supplementary Fig. 16. ^{1}H NMR spectrum of copolymer **3**.

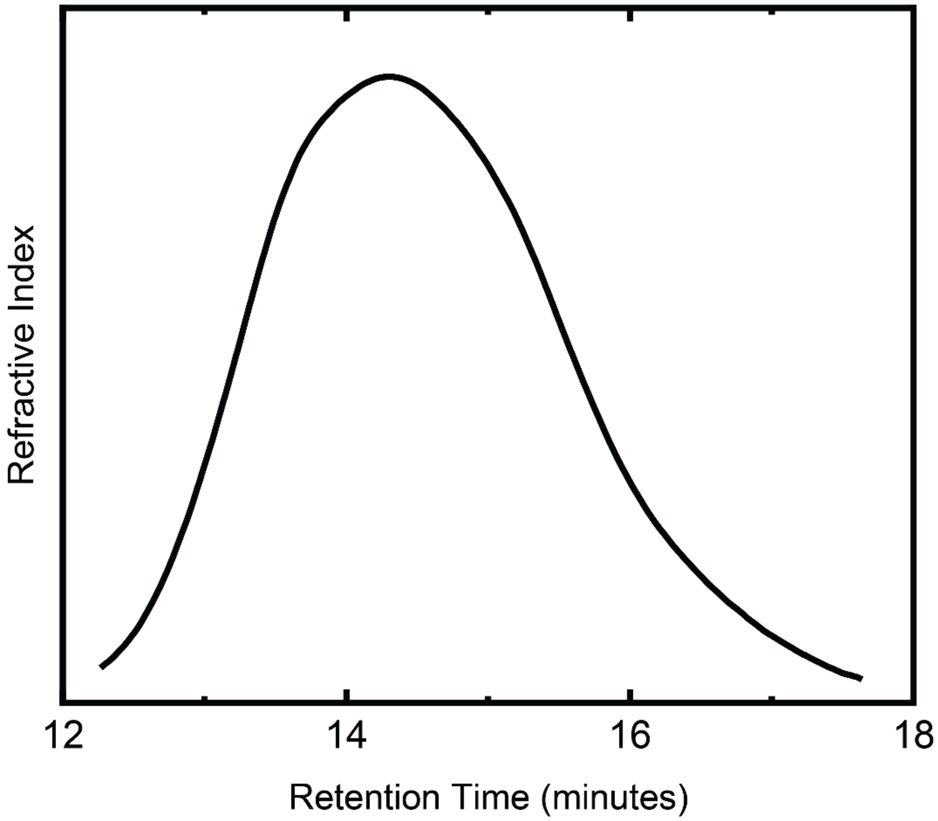

Supplementary Fig. 17. GPC trace of copolymer **1**.

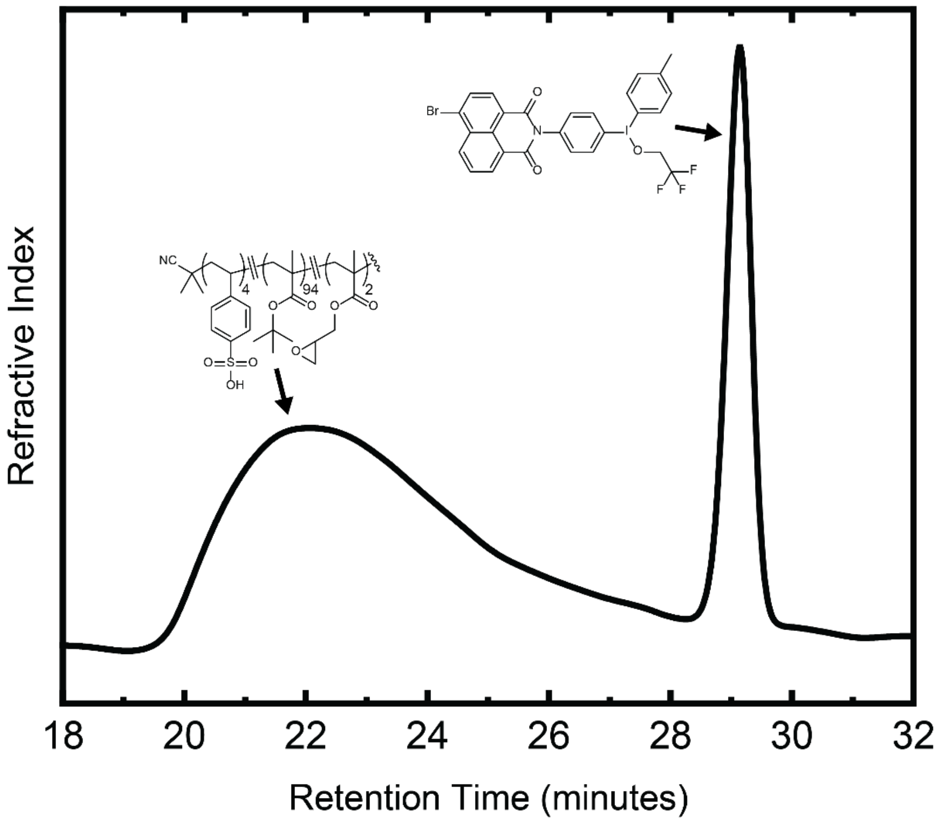

Supplementary Fig. 18. GPC trace of copolymer **2**.

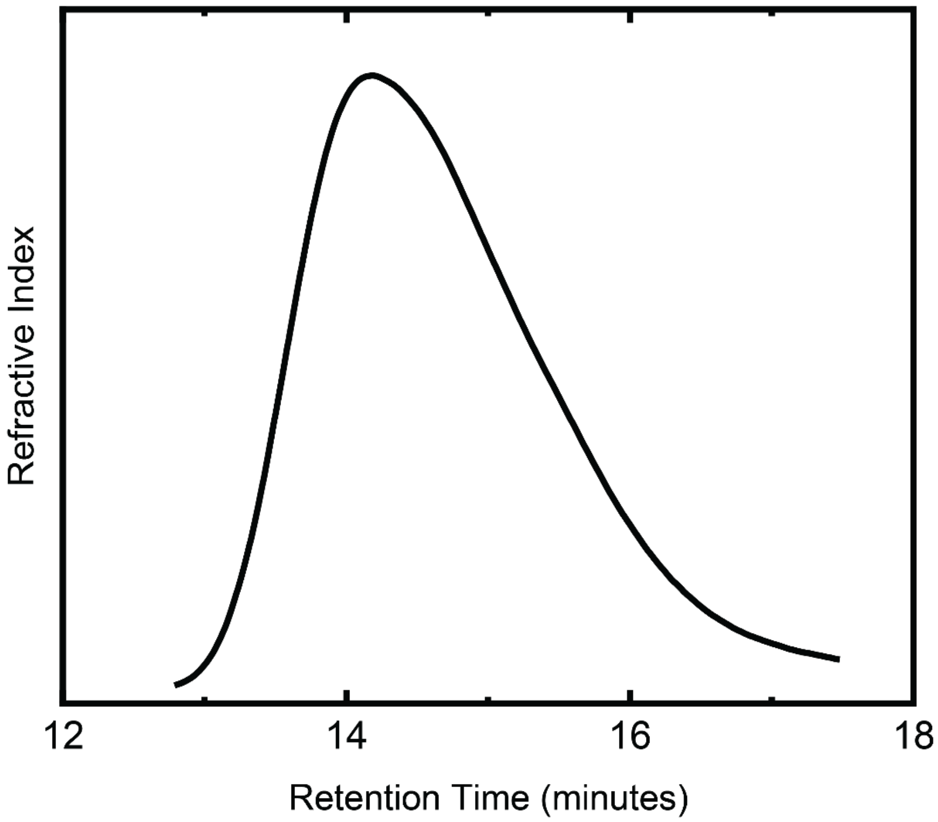

Supplementary Fig. 19. GPC trace of copolymer **3**.

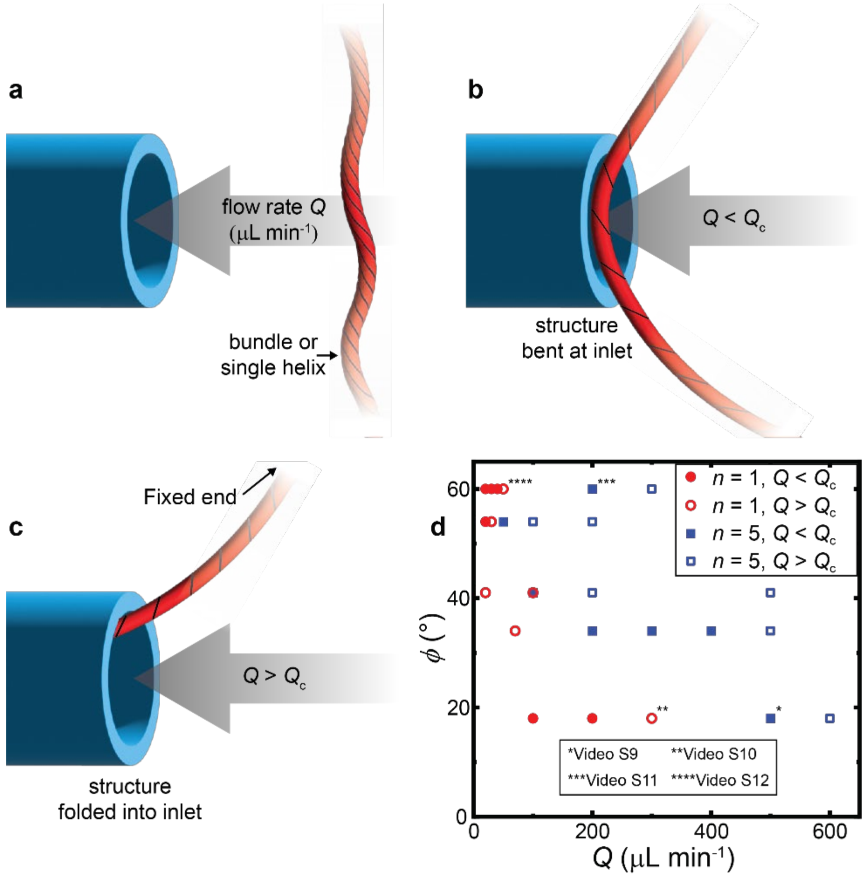

Supplementary Fig. 20. Schematic depiction of the flow bending experiment used to probe helix (n = 1) and bundle (n > 1) mechanics. **a**, a capillary tube connected to a syringe pump is positioned near a bundle or helix of known ϕ and n, with the plane of the inlet roughly perpendicular to the helix/bundle axis. The buffer solution is withdrawn into the capillary tube at a volumetric flow rate Q; **b**, viscous forces draw the helix/bundle to span the capillary inlet. Below a critical flow rate Q_c, the fiber is bent by the flow and pinned in place without being fully drawn into the capillary interior. **c**, when the flow rate exceeds Q_c, viscous forces are sufficient to fold the fiber on itself and the fiber is drawn into the capillary interior. By keeping one end fixed to the substrate surface, the helix/bundle can be repeatedly drawn into and expelled from the capillary tube; **d**, a phase map shows $Q < Q_c$ and $Q > Q_c$ measurements for ϕ = 18, 34, 41, 54, and 60°, n = 1 and 5, and 0 < Q < 600 μL min^{-1}.

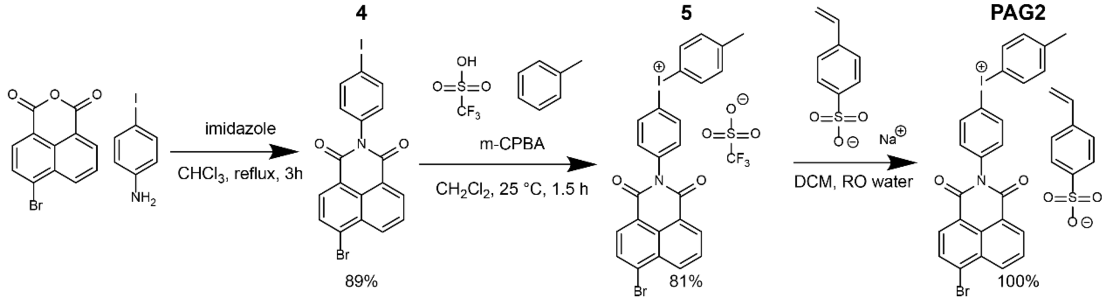

Supplementary Fig. 21. Synthesis of near UV-active styrenic **PAG2** monomer from 4-bromo-1,8-naphthalic anhydride and 4-iodoaniline to molecule **4**, molecule **5** and finally **PAG2**.

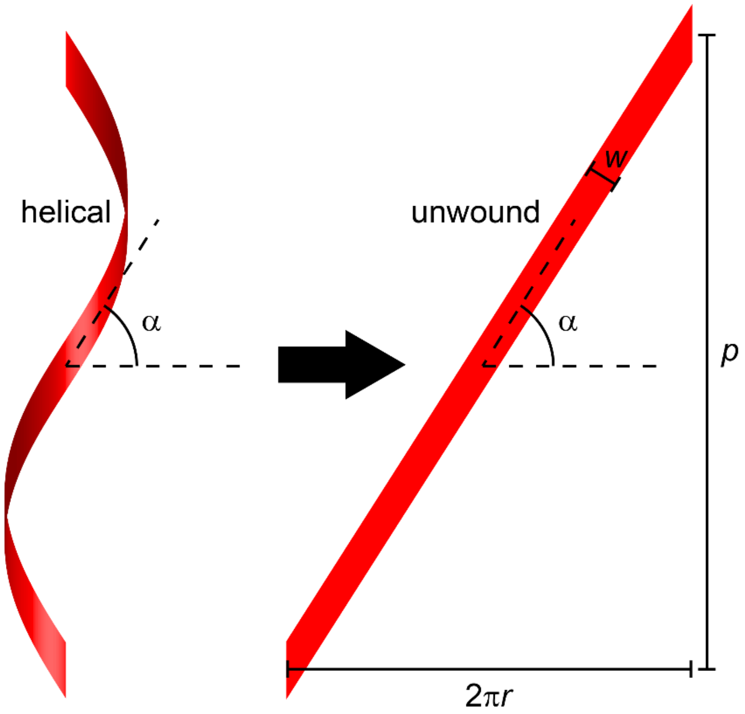

Supplementary Fig. 22. A single helical repeat of a twisted ribbon with width *w*, pitch *p*, radius *r*, and helix angle α, unwound to visualize the relationships between each variable and support the derivation for the contour plot determination of an n-ply helical bundle described in the methods section of the main text.

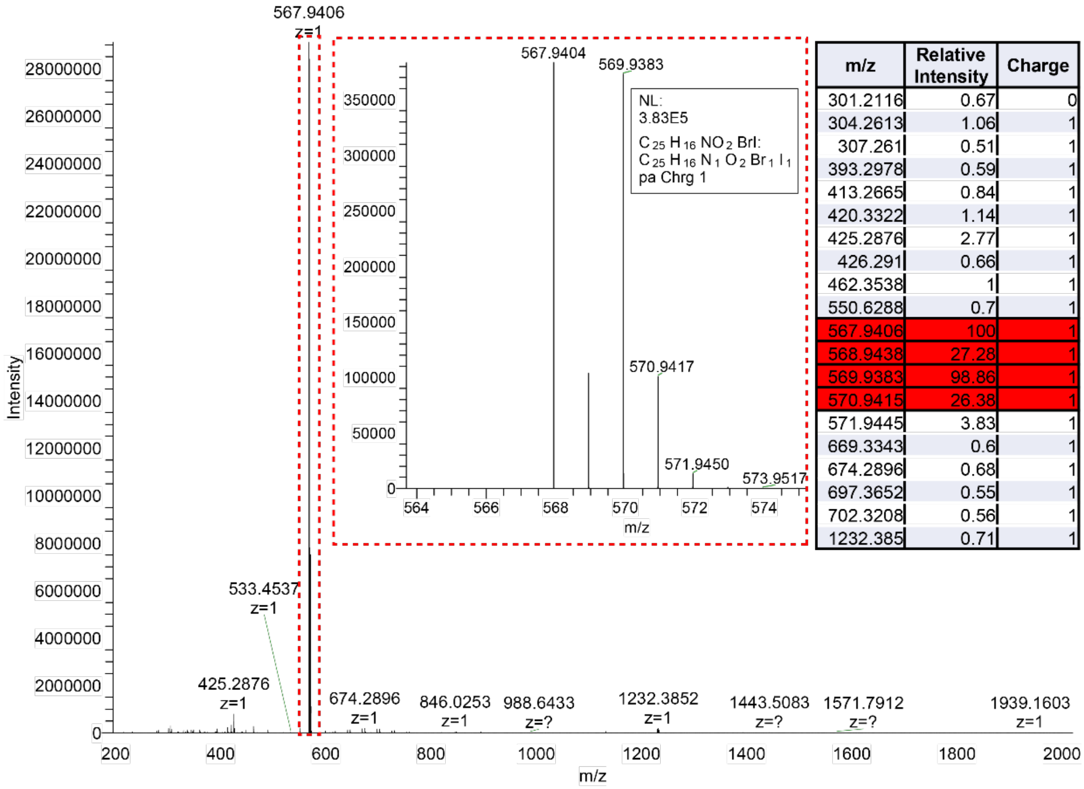

m/z	Relative Intensity	Charge
301.2116	0.67	0
304.2613	1.06	1
307.261	0.51	1
393.2978	0.59	1
413.2665	0.84	1
420.3322	1.14	1
425.2876	2.77	1
426.291	0.66	1
462.3538	1	1
550.6288	0.7	1
567.9406	100	1
568.9438	27.28	1
569.9383	98.86	1
570.9415	26.38	1
571.9445	3.83	1
669.3343	0.6	1
674.2896	0.68	1
697.3652	0.55	1
702.3208	0.56	1
1232.385	0.71	1

Supplementary Fig. 23. High-resolution ESI mass spectrometry data for **PAG2** in positive ion mode. Left inset: the same spectrum spanning 564-574 grams per mole, highlighting the characteristic four-peak fingerprint of the target iodonium cation. Right inset; the twenty most abundant peaks in the spectrum, with target peaks highlighted in red.

Supplementary Note 1: Encoding chirality.

Having demonstrated the power of photocreases of uniform tilt angle to impose a roughly constant pitch and radius (and therefore constant twist and curvature) along the MSP length, we next demonstrate control over the *handedness*. For example, two samples were prepared with h_0 ~ 1 μm and opposing, constant tilt angles (i.e. $\phi_1 \sim -\phi_2$), then released into a pH 8 buffer solution. Supplementary Fig. 6a shows a representative photocreased MSP with ϕ = 48° before (top, imaged through glass substrate), and after (bottom, confocal z-stack) release and coiling into a *left-handed* helix (LHH, bottom). Supplementary Fig. 6b shows the corresponding data for ϕ = -47°, in which photocrease bending affords a *right-handed helix* (RHH). Critically, the bending direction is determined by comparing helix chirality to the photocrease orientation in the substrate-bound state. Specifically, the face of the MSP in contact with the substrate before release, the 'bottom' face, forms the *interior* of the helix (i.e. *bottom-face-in* bending). Accordingly, the LHH and RHH observed in Supplementary Fig. 6a and b, respectively, mean that the photocreases in both cases bend bottom-face-in (schematically depicted in Supplementary Fig. 6c). Every observed helix from the ϕ = 48° experiment was left-handed, while every helix from the ϕ = -47° experiment was right-handed. Moreover, the 33 helices from the experiments of Fig. 3 and hundreds more evaluated microscopically all adopted this bending direction, confirming our ability to control the chirality of MSP coiling with high fidelity.

Supplementary Fig. 6d confirms the chirality of the helix in Supplementary Fig. 6a by showing an axial cross-section (rectangle with dashed border) at 3 successive points along the helix axis, labelled 1-3. For each cross-sectional slice shown in A, an axial perspective with the remaining helix length in the background (top) and the same axial perspective revealing a thin slice of the helix (bottom) are visible. As the cross-section advances along the helix axis (viewing angle indicated by the arrow in Supplementary Fig. 6a), successive segments map a counterclockwise rotation (white arrows), confirming that the helix is left-handed. Supplementary Fig. 6e performs the same analysis for the helix of Supplementary Fig. 6b, verifying that it is right-handed and consistent with the proposed 'bottom-face-in' architecture. The same analyses are highlighted by Supplementary Movies 1-2.

Supplementary Note 2: Estimating Δt and τ.

MSP release is mediated by dissolution of the underlying sacrificial layer, a process that is initiated upon contact with the aqueous solution front traversing a sample. Δt is the interval spanning the release initiation of two adjacent MSPs, i.e., the time required for the aqueous front to cover inter-MSP spacing *d*. In the experiments described here, the largest value of *d* is 60 μm and the solvent front speed can be estimated by MSP release movies. The bundle of Fig. 4a is a representative example, with frame width ~4.260 mm and the solvent front is visible for 200 ms (2 frames at 10 fps), affording an approximate speed of 21.3 mm s^{-1}. $\Delta t = d/(21.3 \text{ mm s}^{-1}) = 2.8\text{x}10^{-3}$ s, while τ, the characteristic timescale of bundling, is ~10 s (estimated by the time interval required for the bundle to stop spinning after initial contact with the aqueous front). Thus $\tau \sim 3500\Delta t$ for the experimental conditions used in this work.

Supplementary Note 3: Rationale for monomer ratios in copolymers 1 and 2.

Copolymers were prepared with the highest possible density of *t*-butyl ester pendent groups to offer the greatest contrast in polarity between the masked (hydropohobic) segments and the irradiated (swellable and hydrophilic) photocreases. This was desirable because it localized any swelling-mediated deformation within the photocreases. Thus, copolymers **1** and **2** contain ~94 mol% of *t*-butyl methacrylate, while copolymer **3** is a *t*-butyl methacrylate homopolymer (with 0.2 mol% rhodamine B methacrylate) to be used as a PAG-free control. The PAG monomers are the second-most abundant monomer in the backbone. During initial polymer formulation, it was found that deprotection of pendent *t*-butyl esters only occurred when [PAG] > [glycidyl methacrylate] (GMA), suggesting that epoxide moieties preferentially scavenge photoacid. ~2 mol% of GMA afforded sufficient crosslinking to afford water-swellable domains in irradiated MSPs and films, and a 2:1 ratio of [PAG]:[GMA] was chosen to ensure that sufficient photoacid was available to drive epoxide crosslinking *and* deprotection of *t*-butyl esters.

Supplementary Note 4: Comparing helix and bundle mechanics by bending in flow.

To gain some insight into the elastic behavior of helices (n = 1) versus bundles across a range of photocrease angles ϕ, five samples were prepared, each with i) an MSP array with n = 5, and ii) a separate, parallel MSP on the same substrate (n = 1). The 5 samples were patterned with ϕ = 18, 34, 41, 54, and 60°,

then released into pH 8 buffer in 3 mM sodium dodecyl sulfate. Upon release, each sample contained a bundle of n = 5 and a helix of n = 1. For each coiled structure (5 helices and 5 bundles), a capillary tube (inner radius ~350 μm) was connected to a syringe pump and positioned perpendicular to the long axis of the coil. Then, the syringe pump was programmed to withdraw solution at a flow rate $0 \leq Q \leq 600$ μL min^{-1}, creating a flow into the capillary inlet (Supplementary Figure 20a). At low flow rates, the coiled structure was pressed by the flowing solution against the capillary inlet, but the volumetric flow rate (Q) was less than the critical rate (Q_c) required to fold the helix/bundle back on itself and enable suction into the capillary interior (Supplementary Fig. 20b). However, as Q increased, helices/bundles bent farther into the capillary inlet; at a sufficiently high flow rate ($Q > Q_c$), the helix/bundle bent at a radius < 350 μm, enabling complete withdrawal into the capillary interior (Supplementary Fig. 20c). Supplementary Fig 20d maps volumetric flow rates below (filled) and above (hollow) Q_c for helices (n = 1, circular data points) and bundles (n = 5, square data points) at each representative value of ϕ, affording two main takeaways. First, bundling increases Q_c across all measured values of ϕ, confirming that bundling increases stiffness in bending. Second, Q_c decreases dramatically with increasing ϕ for $18° \leq \phi \leq 54°$ in both the n = 1 and n = 5 cases suggesting ready tunability of bending stiffness as a function of programmable helix angle in individual helices and in bundles. Interestingly, increasing ϕ further to 60° afforded an *increase* in bending stiffness, possibly attributable to an overlapping effect between successive coils. A more thorough analysis of mechanical properties in tension, including analysis of the fiber gripping effect that allows spun fibers to bear load over lengths exceeding L for any constituent filament, is ongoing and exceeds the scope of this manuscript.

Supplementary Movie 1.
Scanning the length of the helix in Supplementary Figure 6a,d; counterclockwise rotation confirms left-handed chirality.

Supplementary Movie 2.
Scanning the length of the helix in Supplementary Figure 6b,e; clockwise rotation confirms right-handed chirality.

Supplementary Movie 3.
A coiling array of copolymer **2** MSPs in pH 8 buffer solution with photocrease dose of 25 J cm^{-2} (λ_{max} = 365 nm). Scale bar 500 μm. 1x playback speed.

Supplementary Movie 4.

A coiling array of copolymer **2** MSPs in pH 8 buffer solution with photocrease dose of 75 J cm^{-2} (λ_{max} = 365 nm). Scale bar 500 μm. 1x playback speed.

Supplementary Movie 5.

Deflection of copolymer **2** MSPs of h_0 ~ 1 μm without photopatterning and after release into aqueous solution; MSPs do not spontaneously coil and are elastically bent in flow. Scale bar 500 μm. 1x playback speed.

Supplementary Movie 6.

Deflection of copolymer **3** MSPs of h_0 ~ 1 μm after patterning with photocrease tilt angle ~ 45° and release into pH 8 buffer solution; MSPs do not spontaneously coil and are elastically bent in flow. Scale bar 200 μm. 1x playback speed.

Supplementary Movie 7.

Release and coiling of a photocreased MSP array in pH 8 buffer with d = 60 μm and ϕ = 26°. Scale bar 500 μm. 1x playback speed.

Supplementary Movie 8.

Release and coiling of a photocreased MSP array in pH 8 buffer with d = 60 μm and ϕ = 44°. Scale bar 500 μm. 1x playback speed.

Supplementary Movie 9.

Bending a 5-MSP bundle (ϕ = 18° and $h_{0,avg}$ = 0.84 μm) in pH 8 buffer with 3 mM sodium dodecyl sulfate via suction at 500 μL min^{-1} through a capillary tube. Scale bar 1 mm. 5x playback speed.

Supplementary Movie 10.

Bending a single MSP helix (ϕ = 18° and and h_0 = 0.98 μm) in pH 8 buffer with 3 mM sodium dodecyl sulfate via suction at 300 μL min^{-1} through a capillary tube. Scale bar 1 mm. 5x playback speed.

Supplementary Movie 11.

Bending a 5-MSP bundle (ϕ = 60° and $h_{0,avg}$ = 1.04 μm) in pH 8 buffer with 3 mM sodium dodecyl sulfate via suction at 200 μL min^{-1} through a capillary tube. Scale bar 1 mm. 5x playback speed.

Supplementary Movie 12.

Bending a single MSP helix (ϕ = 60° and and h_0 = 1.10 μm) in pH 8 buffer with 3 mM sodium dodecyl sulfate via suction at 30, 40, and finally 50 μL min^{-1} through a capillary tube. Scale bar 1 mm. 5x playback speed.
