# Supplementary material for: Self-spinning filaments for autonomously linked microfibers
Source: Nat Commun. 2023 Feb 4;14:625. doi: 10.1038/s41467-023-36355-w (PMC9899204; doi:10.1038/s41467-023-36355-w)
Supplement: Supplementary file 3 — Description of Additional Supplementary Files [file 41467_2023_36355_MOESM3_ESM.pdf]

Description of Additional Supplementary Files

Supplementary Movie 1.
Scanning the length of the helix in Supplementary Figure 6a,d; counterclockwise rotation confirms left-handed chirality.

Supplementary Movie 2.
Scanning the length of the helix in Supplementary Figure 6b,e; clockwise rotation confirms right-handed chirality.

Supplementary Movie 3.
A coiling array of copolymer **2** MSPs in pH 8 buffer solution with photocrease dose of 25 J cm^{-2} (λ_{max} = 365 nm). Scale bar 500 μm. 1x playback speed.

Supplementary Movie 4.
A coiling array of copolymer **2** MSPs in pH 8 buffer solution with photocrease dose of 75 J cm^{-2} (λ_{max} = 365 nm). Scale bar 500 μm. 1x playback speed.

Supplementary Movie 5.
Deflection of copolymer **2** MSPs of h_0 ~ 1 μm without photopatterning and after release into aqueous solution; MSPs do not spontaneously coil and are elastically bent in flow. Scale bar 500 μm. 1x playback speed.

Supplementary Movie 6.
Deflection of copolymer **3** MSPs of h_0 ~ 1 μm after patterning with photocrease tilt angle ~ 45° and release into pH 8 buffer solution; MSPs do not spontaneously coil and are elastically bent in flow. Scale bar 200 μm. 1x playback speed.

Supplementary Movie 7.
Release and coiling of a photocreased MSP array in pH 8 buffer with d = 60 μm and ϕ = 26°. Scale bar 500 μm. 1x playback speed.

Supplementary Movie 8.
Release and coiling of a photocreased MSP array in pH 8 buffer with d = 60 μm and ϕ = 44°. Scale bar 500 μm. 1x playback speed.

Supplementary Movie 9.
Bending a 5-MSP bundle (ϕ = 18° and $h_{0,avg}$ = 0.84 μm) in pH 8 buffer with 3 mM sodium dodecyl sulfate via suction at 500 μL min^{-1} through a capillary tube. Scale bar 1 mm. 5x playback speed.

Supplementary Movie 10.
Bending a single MSP helix (ϕ = 18° and and h_0 = 0.98 μm) in pH 8 buffer with 3 mM sodium dodecyl sulfate via suction at 300 μL min^{-1} through a capillary tube. Scale bar 1 mm. 5x playback speed.

Supplementary Movie 11.

Bending a 5-MSP bundle (ϕ = 60° and $h_{0,avg}$ = 1.04 μm) in pH 8 buffer with 3 mM sodium dodecyl sulfate via suction at 200 μL min^{-1} through a capillary tube. Scale bar 1 mm. 5x playback speed.

Supplementary Movie 12.
Bending a single MSP helix (ϕ = 60° and and h_0 = 1.10 μm) in pH 8 buffer with 3 mM sodium dodecyl sulfate via suction at 30, 40, and finally 50 μL min^{-1} through a capillary tube. Scale bar 1 mm. 5x playback speed.
